# Supplementary material for: Wing Plasticity Is Associated with Growth and Energy Metabolism in Two Color Morphs of the Pea Aphid
Source: Insects. 2024 Apr 16;15(4):279. doi: 10.3390/insects15040279 (PMC11050552; doi:10.3390/insects15040279)
Supplement: Supplementary file 1 [file insects-15-00279-s001.zip › Supplementary Figure S1-2.pdf]

Supplementary Materials for

Wing Plasticity Is Associated with Growth and Energy Metabolism in Two Color Morphs of the Pea Aphid

Hehe Cao, Xi Wang, Jiawei Wang, Zhaozhi Lu, and Tongxian Liu

Corresponding authors: zhaozhi@qau.edu.cn (Z.L.); tx.liu@gzu.edu.cn (T.L.)

The PDF file includes:

Figures S1 and S2

Other Supplementary Material for this manuscript includes the following:

Table S1 All differentially expressed genes between red and green morph aphids (Excel format)

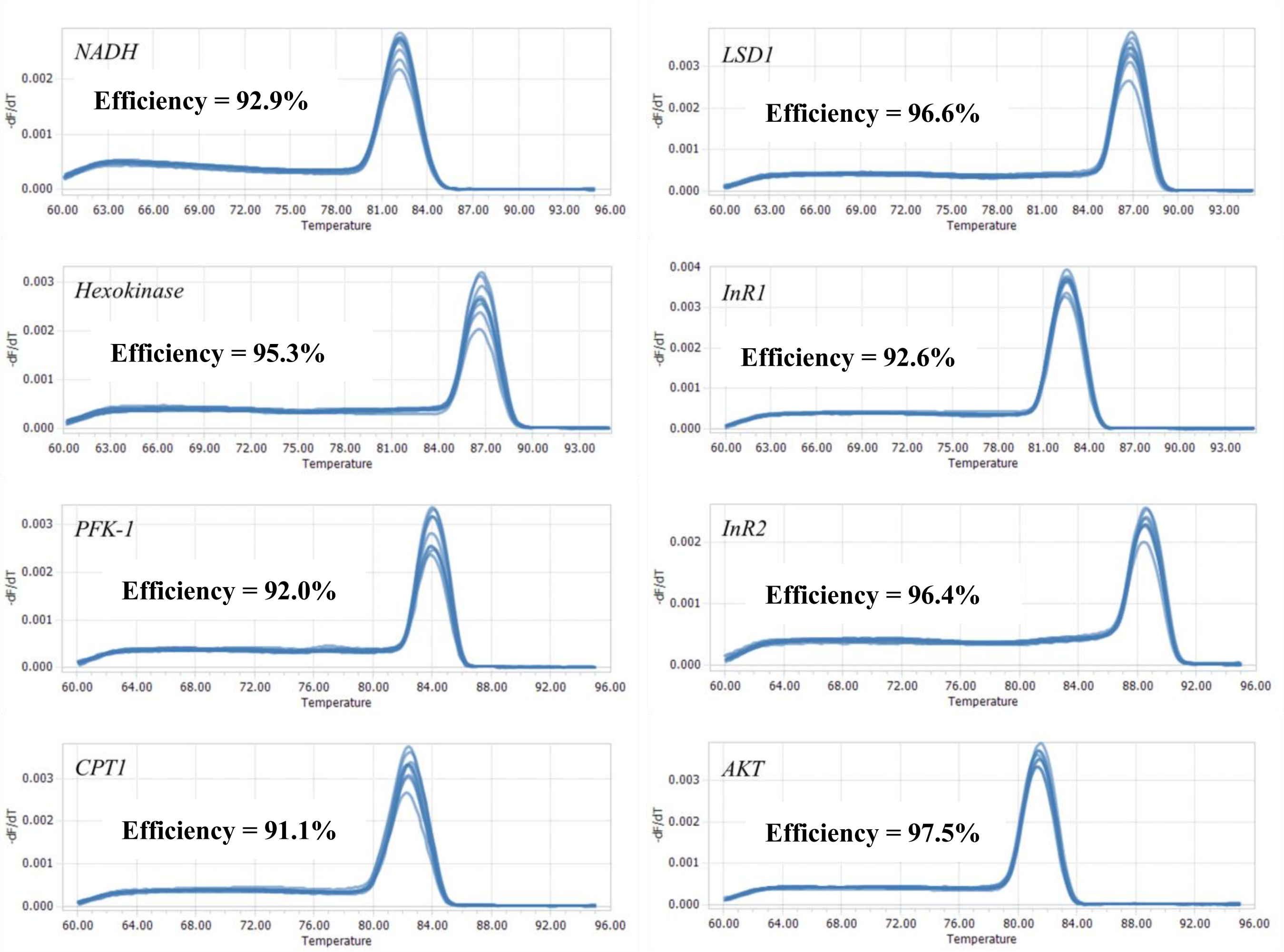

Figure S1. Amplification efficiency and melt curves for primers used in this study.

The amplification efficiency ranges from 91.1% to 97.5%, suggesting that the PCR efficiencies for primers is suitable. The melt curve for each gene has only one peak, suggesting that the primers is specific for amplification.

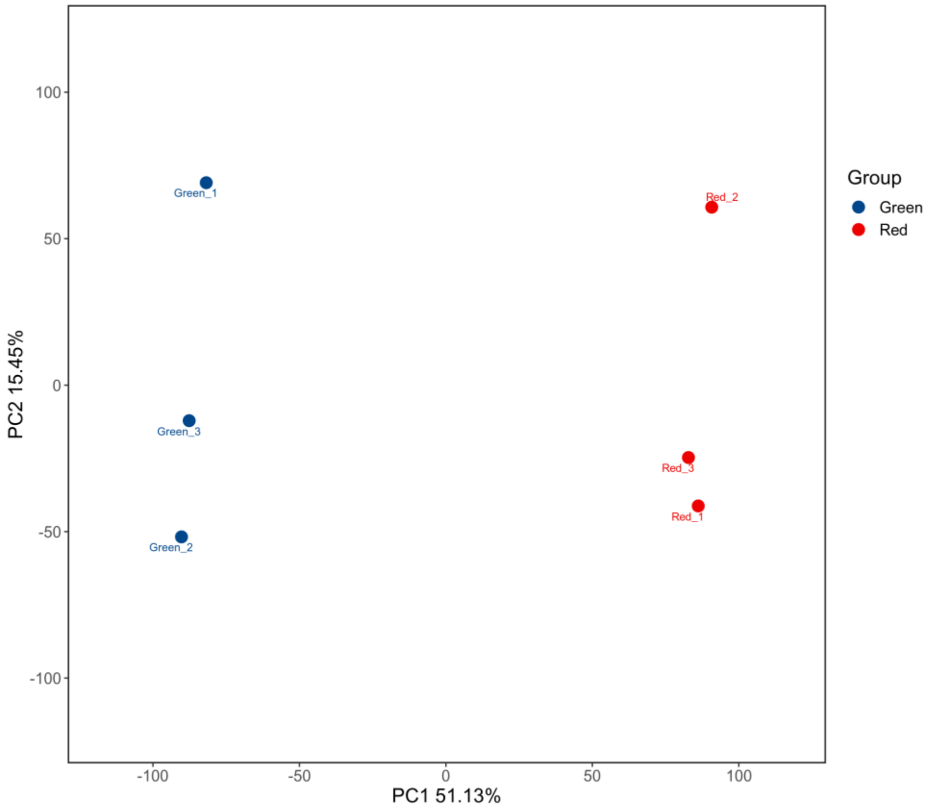

Figure S2. Principal component analysis (PCA) of all differentially expressed genes between green and red morph aphids. Principal component axis 1 explained ~51% of the variance, indicated a large effect of aphid color on gene expression. Principal component axis 2 exhibits 15% of the variance, suggested that different samples within each color aphid had a small variance.
